# Extracellular Vesicles Involvement in the Modulation of the Glioblastoma Environment

**DOI:** 10.1155/2020/3961735

**Published:** 2020-01-28

**Authors:** Fausta Ciccocioppo, Paola Lanuti, Marco Marchisio, Sebastiano Miscia

**Affiliations:** ^1^Department of Medicine and Aging Sciences, University “G. D'Annunzio” Chieti-Pescara, Chieti, Italy; ^2^Center on Aging Science and Translational Medicine (Center for Advanced Studies and Technology (CAST)), University “G. D'Annunzio” Chieti-Pescara, Chieti, Italy

## Abstract

Glioblastoma (GBM) is the most deadly primary brain tumour and is a paradigmatic example of heterogeneous cancer. Although expanding data propose the phenotypic plasticity exhibited by glioblastoma cells, as a critical feature involved in the tumour development and posttherapy recurrence, the central machinery responsible for their aggressiveness remains elusive. Despite decades of research, the complex biology of the glioblastoma is still unknown. Progress in genetic and epigenetic discoveries has improved diagnostic classification, prognostic information, and therapeutic planning. In the complex model of intercellular signalling, several studies have shown that extracellular vesicles have a key role in the intercellular communication among GBM cells and the tumour microenvironment modulation. The purpose of this review is to summarize the role of the EV-mediated intercellular crosstalk in the glioblastoma physiopathology.

## 1. Introduction

Glioblastoma (GBM) is the most common and malignant brain tumour in adults [1]. Despite aggressive treatments used in GBM, such as surgical resection followed by radiotherapy and temozolomide therapy and in tumour regrowth/recurrence, salvage therapy options, including repeat surgical resection, antiangiogenic therapy (bevacizumab), and immunotherapy, the limited success of current treatment leads to poor prognosis [2].

Growing data propose the phenotypic plasticity of the glioblastoma cells as a critical feature involved in the tumour growth and posttherapy recurrence. Anyway, the mechanisms responsible for the glioblastoma aggressiveness remain still unknown [3]. The GBM therapy resistance is linked both to intrinsically aggressive traits as well as to its heterogeneity [2]. Such heterogeneity derives not only from the accumulation of mutations but also from the dynamic changes in the cell status, stemming from the clonal genomic selection and the phenotypic modifications [4, 5]. In reason of the highest deadly index in GBM, the clarification of the physiopathology of the tumour heterogeneity is of relevance for the development of new therapeutic approaches in the perspective of personalized medicine.

Glioblastoma is a paradigmatic example of heterogeneous tumours, in which although partially elucidated, genetics profile and molecular analyses have provided significant information, improving its subtyped classification, as well as its prognostic evaluation and new targeted therapies [2]. In particular, a better understanding of the molecular pathways that drive GBM aggressive features has led to the assessment of therapeutic agents able to target tumour cells. The so-called targeted anticancer therapies, including therapies targeting tumour growth factor receptors and downstream pathways, cell cycle and epigenetic regulation, and angiogenesis and antitumor immune response, are under evaluation as single agents or in combinations [6, 7]. On the other hand, aside from the heterogeneity linked to distinct genetic mutation, studies on GBM stem-like cells have shown that the functional cell diversification can also be described within an unchanged genomic background [3, 8].

Intriguingly, GBM cells have shown specific transcriptomic patterns associated with distinctive stem-like phenotypes and tumorigenic behaviours reactive to environmental signature [9].

In line with the data mentioned above, several studies have shown that the extracellular vesicles, a novel signalling complex with multifaceted functions in neuronal and glial cross-talk, have a crucial role in the communication among cell subtypes into GBM and in the tumour microenvironment modulation [10].

The purpose of this review is to summarize the EV role in the intercellular crosstalk, underling the advances in the knowledge of genetic profiling and epigenetic plasticity in the glioblastoma physiopathology.

## 2. Genetic Profile and Epigenetic Plasticity in Glioblastoma

Genetic analysis of tumours has provided an innovative approach for the diagnosis of glioblastoma.

Different molecular classes of gliomas have been recognized and revised by the fourth edition of the internationally accepted World Health Organization (WHO) Classification of CNS Tumors published in 2016, which has announced the new guidelines for the diagnosis of glial tumours, focusing on the use of genetic profile in the glioma subclassification (Table 1). These include the mutation of the Isocitrate Dehydrogenase 1 and 2 genes (IDH1/2), the codeletion status of chromosome arms 1p and 19q, and histone 3 mutational status, able to distinguish biologically and clinically glioma behaviour [19].

Several studies have proposed that specific genetic profiles create complex signaling networks linked to cancer beginning and spread, providing better information to guide the GBM diagnosis, therapeutic approach, and prognosis [2, 20].

Genome-wide expression profiling and global DNA methylation analysis have separated adult GBMs into two groups, IDH wild-type and IDH mutant, providing new clinical tools.

The molecular profile associated with the clinical outcome has proposed IDH as an essential prognostic biomarker in brain tumours. The IDH is an enzyme involved in cellular metabolism, which is explicitly designated as a hallmark of the “reprogramming cellular energetics” mechanisms [21]. The IDH mutations are frequently described in the secondary GBM that arise in a preexisting low-grade lesion and show a better prognosis than IDH wild-type GBMs [2, 13].

Distinctive genetic hallmark in the primary GBMs are represented by amplification of epidermal growth factor receptor (EGFR) and phosphatase and tensin homolog (PTEN) tumour suppressor gene and loss of other cyclin-dependent kinases, lacking IDH mutations [22].

Another distinctive entity of high-grade midline pediatric gliomas, lacking IDH mutations, is a glioma H3 K27 M mutant associated with mutations of the gene encoding histone H3 [23].

Low-grade tumours, such as astrocytoma and oligodendroglioma, are genetically distinct entities; both entities share high frequencies of IDH mutations. The isocitrate dehydrogenase mutation and loss of portions of chromosomes 1 and 19 (1p/19q codeletion) are described in the oligodendrogliomas; on the contrary, mutations of *α*-thalassemia/mental retardation X-linked protein (ATRX) and tumour protein P53 (TP53) are frequent in the astrocytomas [24, 25].

In addition, mutations in a tumour suppressor gene in the mitogen-activated protein kinase pathway (BRAF) is described in a group of low-grade tumours, including pilocytic astrocytoma, ganglioglioma pleomorphic xanthoastrocytoma, and subependymal giant cell tumours [26].

Thus, the isocitrate dehydrogenase status, such as IDH-1 and IDH-2 mutations, and ATRX mutation or loss and tumours with 1p19q co-deletion has been associated with better prognosis [27].

Another positive prognostic GBM marker is hypermethylation of O-6-methylguanine-DNA-methyltransferase (MGMT) promoter that is associated with better response to combined radiation-temozolomide therapy [2, 28]. On the other hand, mutations in the promoter for telomerase reverse transcriptase (TERT), as well as increased amounts of Ki-67, have been described as a worse prognostic in both IDH mutant and IDH wild-type GBMs [12, 29].

Genetic information represents clinical tools that, together with image interpretation, reducing the number of miscategorized tumours, improve the GBM patient management.

In the glioblastoma, the cells are likely to undergo epigenetic changes, acquiring stem cell features. Expanding data suggest that epigenetic plasticity is linked to GBM stem-like cell adaptations to their changing microenvironment [30].

Posttranslational histone modifications, such as acetylation, phosphorylation, methylation, or ubiquitinylation of histone arginine (R) and lysine (K), are proposed as critically linked to epigenetic plasticity in the GBM [3].

These histone changes are associated with transcriptional modifications due to abnormal DNA structural accessibility linked to the altered affinity between DNA and histones, as well as to new binding sites for chromatin remodelling factors [31].

Epigenomic studies in the brain have confirmed emerging data on the link between histone H3 K4 and K27 trimethylation (H3K4me3 and H3K27me3) with transcriptional expression and repression, respectively, in embryonic stem cells (ESC) [32, 33].

An interesting study has described the H3K4me3 and H3K27me3 as a mark of the glioblastoma cell tumorigenicity linked to the network of the transcription factors [3], pointing the involvement of the hypoxia-inducing factor (HIF) family member aryl hydrocarbon receptor nuclear translocator 2 (ARNT2) in the modulation glioblastoma cell aggressiveness. Notably, they have demonstrated that ARNT2 controls the expression of several transcription factors associated with the stem-like properties of glioblastoma cells and is essential for full tumorigenicity of glioblastoma cells [3].

## 3. Role of the Extracellular Vesicles in the Cross-Talk among Glioblastoma Cells

Extracellular vesicles (EVs), referred as membrane-bound organelles virtually released by all cell types, have emerged as a novel signalling model with multiple functions in neuronal and glial cross-talk, thus promoting microglia-mediated immune surveillance, cellular proliferation, differentiation, senescence, and plasticity [34–39]. Extracellular vesicles, which range from 30 to 1000 nm in size, include a spectrum of vesicles, such as exosomes, microvesicles, and apoptotic bodies, which are categorized according to their diameters, biogenesis, morphology, and cargo [40–42].

Exosomes are small vesicles (approximately 50–100 nm in diameter) released by exocytosis of multivesicular bodies (MVBs) and surrounded by a phospholipid bilayer that exposes phosphatidylserine on their surfaces. The common exosome markers are CD63, CD81, CD9, LAMP1, and TSG101 [43, 44]. Among different ways, exosomes exert their biological functions, including direct surface contact between EVs and target cells, their endocytosis, EV-cell membrane fusion, and horizontal transfer of mRNA/miRNA, oncogenic receptors, and HIV particles [40–43]. Of interest, the major transforming event occurring in the 50% of GBM cases is the amplification, rearrangement, or point mutations of the oncogenic receptor tyrosine kinases, known as EGFR, whereas a large proportion of cases is positive for a distinct mutant known as EGFRvIII [45]. Oncogenic receptors, including the EGFRvIII, often reside into regions of the plasma membrane, from which extracellular vesicles originate in cancer cells (lipid rafts), and as a result, they could themselves be integrated into the vesicle cargo. In this contest, several recent studies have described the intercellular transfer of this oncogenic receptor by extracellular vesicles derived from glioma cells [45, 46].

Exosomes have mainly been proposed both as mediators of immune cell functions (involving dendritic and T and B cells, as well as macrophages) and in tumour mechanisms, where their key roles are linked to antigen presentation as well as to immunomodulatory activities [47, 48].

Microvesicles are vesicles of the 100–1,000 nm in diameter [43, 48] and are surrounded by a phospholipid bilayer that may or not expose phosphatidylserine on the membrane surface [49]. They are described as endothelial, platelet, and red blood cell products and originate by budding/blabbing of the plasma membrane after activation of cell surface receptors [50, 51]. Microvesicles have procoagulant roles and represent a form of secretion for IL1b. The function of MVs has also been proposed in the rheumatoid arthritis pathogenesis, as well as in the mechanisms associated with tumour proinvasive characteristics and in the induction of oncogenic cellular transformation and fetomaternal communication [43, 52, 53].

Finally, apoptotic bodies (about 1–5 *μ*m in diameter) are released as blebs from cells undergoing apoptosis and are characterized by phosphatidylserine externalization [54, 55]. Apoptotic bodies horizontally transfer oncogenes and/or DNA and are involved in the presentation of T cell epitopes upon their uptake by phagocytic cells and in the representation of B cell autoantigens [43].

An international consortium, however, agreed on the use of the term of extracellular vesicles (EVs) to uniform the confused terminology present in the literature [56, 57].

In the Central Nervous System (CNS), extracellular vesicles have been involved in the rich network of intercellular connections that sustain the physiological homeostasis as well as the development of the pathogenic machinery leading to neurological diseases (neurodegenerative disorders, as well as brain tumours and stroke) [10].

Increasing data describe the ability of the EVs released by neurons and glial cells to pass across the brain-blood barrier (BBB), through a mechanism known as transcytosis [58], allowing the systemic propagation of physiopathological data in vivo [40, 59, 60].

Recent researches have proposed that some macromolecules can be transported across the BBB through the process of receptor-mediated transcytosis (RMT) [61]. The RMT has been described in epithelial cells, where following the bind of the ligands to their receptors in the apical membrane, their internalization and intracellular transport to the basolateral membrane occurs [62].

Extracellular vesicles represent an exclusive way of long-range shuttling for a broad spectrum of bioactive molecular cargoes, such as nucleic acids (DNA, mRNA, miRNA, and other noncoding RNAs), proteins (receptors, transcription factors, membrane-bound signalling enzymes, and extracellular matrix-ECM-proteins), and lipids [63–65].

In reason of the EV ability to modify the phenotype of their target cells, they result to be a key mediator in physiological and pathological mechanisms, such as neuronal and glial response to brain injury [35].

Several studies refer to a network of the EVs-mediated cellular interactions linked to cancer spreading [10, 52, 53] with a prion-like model. Extracellular vesicles originated by cancer cells induce tumour-promoting effects in nearby cells [66], releasing not only soluble factor and proteins, such as oncoproteins, ephrins, and chemokine receptors but also DNA, mRNAs, miRNAs, and other small noncoding RNAs that mediate specific signalling machineries related to dysregulated cell growth and hypoxic environment development [45, 67–72]. Recent literature proposed a link between the Ephrin (Eph) family and the extracellular vesicles-mediated signalling pathway [73]. Several studies have analysed the role of the Eph receptor and the corresponding ephrin ligands and how this family of receptors might function to promote the development and the malignant progression of glioma [74, 75]. Eph receptors and ephrin ligands display regulatory functions in the central nervous system development and on several adult brain functions, including the synaptic structure modeling [76]. Besides, there is not evidence on the fact that the Eph/ephrin system has a crucial role in maintaining the neural stem cell niche, in physiological conditions [77]. Notably, the glioma cell invasion process appears to be similar to neuronal cell migration during neural development. So, the infiltrative character of GBM resembles the glial progenitors' movement, suggesting that the overexpression and the deregulation of the Eph/ephrin system are involved in GBM tumorigenesis, participating to GBM invasion, metastasis, and angiogenesis [76, 78].

**Figure 1:**
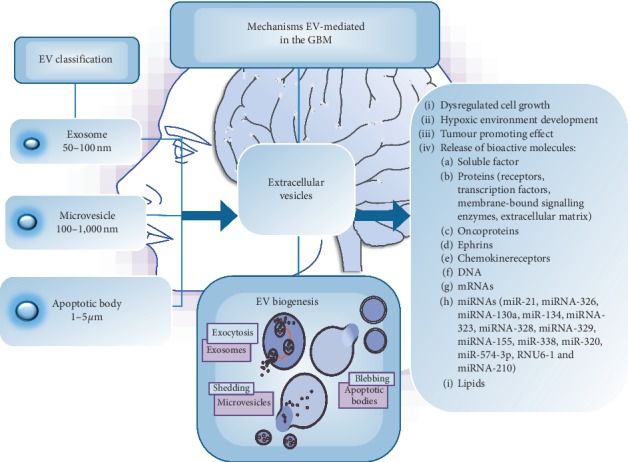
Several studies describe extracellular vesicles as a novel signalling complex involved in the neuronal and glial cross-talk. They have a crucial role in the communication among cell subtypes into GBM and in the tumour microenvironment modulation. Extracellular vesicles represent an exclusive way of long-range shuttling for a broad spectrum of bioactive molecular cargoes, such as nucleic acids, proteins, and lipids.

Among the brain tumours, GBM displays a high cellular heterogeneity, presenting a variety of cell subtypes, such as cancer stem cells, tumour cells and nonneoplastic parenchymal cells, comprising vascular cells, microglia, and peripheral immune cells. Extracellular vesicles have a key role in the communication among these cells [10](Figure 1).

In line with data as mentioned above, several studies have shown that when GBM-derived EVs are put into cocultures with endothelial cells, they induce the alteration in gene expression and angiogenesis through the modulation of endothelial cells [66, 67, 69, 79] and stimulate the proliferation of the cells from which they were originated [70, 80].

Increasing evidence shows that the crucial way of intercellular communication EV mediated among GBM cells is the transfer of specific miRNAs to target cells [81].

The deregulation of miRNAs is a typical GBM feature, linked to tumour suppression and oncogenesis [82].

The microRNAs are a class of small noncoding RNAs of approximately 22 nucleotides in length that are implicated in posttranscriptional regulation of gene expression, silencing the expression of target genes [83, 84]. Numerous studies proposed that aberrant microRNA expression is associated with several pathogenic mechanisms in GBM, representing a prognostic tool in the clinical outcome of GBM patients [85].

The upregulation of miRNA-326 and miRNA-130a, as well as the downregulation of miRNA-323, miRNA-329, miRNA-155, and miRNA-210, have been associated with more prolonged overall survival in GBM patients [86]. Moreover, upregulated levels of miRNA-326 and miRNA-130a related to downregulation of miRNA-155 and miRNA-210 could be linked to extended progression-free survival [85]. On the other hand, Wu et al. have described the downregulation of miRNA-328 related to worse survival in primary GBM [87].

GBM-derived extracellular vesicles are enriched and shuttle specific microRNAs [69]. Thus, this is not a passive process and may play an essential role in the tumour microenvironment modulation [83].

The upregulation of the miR-221 is a biomarker for glioma. Akers et al. have investigated the miRNA pattern in different EV subpopulations [88]. Notably, they described that the miR-21, together with other GBM-pertinent miRNAs, are highly enriched in EVs derived from the cerebrospinal fluid of GBM patients [10, 88]. An interesting study has suggested that EVs derived from glioma stem cells can promote the angiogenic ability of endothelial cells through the miR-21/VEGF/VEGFR2 signal pathway [89].

The miRNA expression was also investigated also in the serum and plasma into the exosomes of GBM patients, revealing a potential role as a diagnostic biomarker for the miR-320, miR-574-3p, and RNU6-1 [68, 90]. An additional study has identified that miR-134 was epigenetically silenced in gliomas. This research validated the hypothesis that miR-134 target gene KRAS, an upstream regulator of ERK and AKT pathways, highlighting the potential role of miR-134 as a therapeutic target in glioma patients [91]. Data on the regulation of the PKM2/*β*-catenin axis in glioma showed that the PKM2 was a potential target of miR-338 in animal models. The MiR-338, a tumour suppressor, results in downregulation in high-grade gliomas. These results confirm that miR-338 suppresses the PKM2/*β*-catenin axis [92].

The EGFRvIII mutant characterizes a clinical subtype of glioma, and recently, the immunomagnetic exosome RNA analysis has revealed that those exosomes were enriched in EGFR/EGFRvIII and quantifying [10]. Finally, Shao et al. (2015) proposed the exosomal mRNA profile as a clinical tool for response to treatment stratification in GBM patients [10, 93].

## 4. Conclusions

Despite decades of research, the complex biology of the glioblastoma is still unknown. Progress in the genetic discoveries has improved diagnostic classification and prognostic information, addressing image interpretation and therapeutic approaches.

However, in spite of multimodal treatment, patients have poor prognosis with a median survival of 14–16 months and 5-year overall survival (OS) of 9.8% [21].

Understanding the biological actors involved in aggressive tumour behaviour but also the heterogeneity of the disease is fundamental in therapeutic planning.

All in all, further enlarged studies focused on extracellular vesicles could propose them as reliable complementary biomarkers and as an intriguing starting point for the development of novel therapeutic strategies, based on EV modulation in GBM patients.

## Figures and Tables

**Table 1:** Genetic alterations in gioblastoma.

Gene	Alteration	Signaling pathway	References
IDH	Mutation	Metabolism	[11]
EGFR	Deletion (EGFRvIII) Mutation Translocation Amplification	RTK signaling	
ATRX	Mutation	Genome integrity	[12]
TP53	Wild-type (no mutations)	Cell cycle pathways	[13]
PTEN	Deletion Mutation	MAPK and PI3K/mTOR signaling pathways	[13]
RB1 CDKN2A	Loss	Rb pathway	[14]
PTPRZ1	Fusion	RTK signaling	[15]
KIT	Amplification Mutation	Growth factor	[6]
PDGFRA	Amplification	Growth factor	[16]
MDM2	Amplification	Cell cycle pathways	[17]
NF1	Deletion Mutation	MAPK and PI3K/mTOR signaling pathways	[16]
FGFR1, FGFR3	Translocation (FGFR3-TACC3)	Growth factor	[6, 18]
MET	Amplification Translocation	Growth factor	[6]
TERT	Promoter mutation	Telomere maintenance	[17]
